# Comparative proteomic analysis of the zebrafish (*Danio rerio*) perivitelline fluid; identifying putative cortical alveoli-associated proteins

**DOI:** 10.1093/biolre/ioag012

**Published:** 2026-01-29

**Authors:** Blake A Lewis, Karen L Reader, Michael W Pankhurst, Caroline W Beck, Mark P Lokman

**Affiliations:** Department of Zoology, Division of Sciences, University of Otago, Dunedin, New Zealand; Department of Pathology, Faculty of Medicine, University of Otago, Dunedin, New Zealand; Department of Anatomy, Faculty of Biomedical Sciences, University of Otago, Dunedin, New Zealand; Department of Zoology, Division of Sciences, University of Otago, Dunedin, New Zealand; Department of Zoology, Division of Sciences, University of Otago, Dunedin, New Zealand

**Keywords:** zebrafish, perivitelline fluid, cortical alveoli, proteome

## Abstract

Upon fertilization, the egg must have the resources to facilitate successful fertilization and regulate zygotic growth prior to the activation of the embryonic genome. A key biomarker of egg activation is the synchronized release of cortical alveoli (CA) from the egg’s cortex into the perivitelline space. Teleost CA are primarily associated with their highly conserved roles in polyspermy prevention. However, several CA-associated proteins with functions related to innate immunity have been isolated. While there is growing evidence that teleost CA have extended biological roles outside of polyspermy prevention, little work has been done toward developing an understanding of their general proteomic composition. Therefore, the aim of this study was to identify proteins which were proportionally over-represented in the zebrafish (*Danio rerio*) perivitelline fluid (PVF) directly following CA exocytosis to identify a candidate list of CA-associated proteins. This study utilized a novel technique for PVF extraction from water-activated eggs shortly following CA exocytosis which negated the use of fixatives, thereby increasing PVF sample integrity for downstream mass-spectrometry analysis. Moreover, this study provides the first comparative proteomic analysis of the PVF relative to the yolk and cytoplasm of the zebrafish egg. As a result, 44 proteins were identified which were proportionally over-represented in the PVF, relative to the yolk and cytosol, of the water-activated unfertilized egg. The resulting PVF proteome was comprised of proteins associated with functions in carbohydrate binding and peptidase regulation. Many of these proteins are compelling candidates for being CA-derived and have been previously implicated in innate immunity.

## Teaser

This study identified proteins which were proportionally over-represented in the perivitelline fluid of zebrafish eggs, with a specific focus on uncovering the maternal contributions to the embryonic microenvironment and identifying putative cortical alveoli-related proteins.

## Graphical Abstract

**Graphical Abstract:**
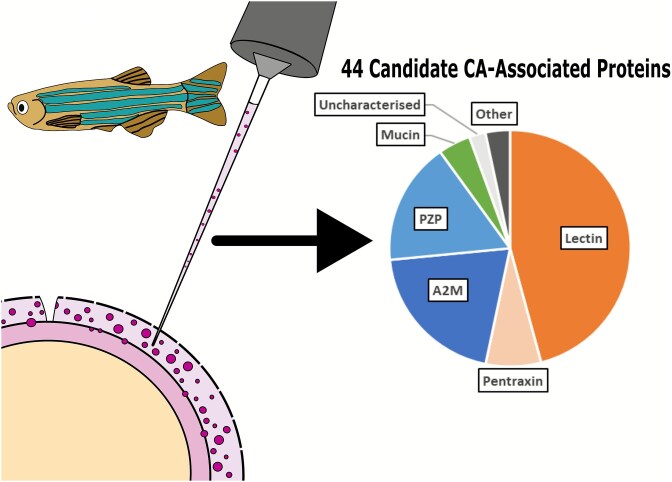

## Introduction

Cortical alveoli (CA) represent a substantial maternal investment in the form of biologically complex, densely packed, glycoprotein-filled secretory vesicles, which are released during a one-off mass exocytosis event at the time of fertilization. The scale of this maternal investment is most clearly demonstrated during the late stages of oocyte primary growth, often referred to as “cortical alveolus stage,” where in zebrafish, the whole ooplasm becomes inundated with newly synthesized CA [1]. The maternal investment into these single-use secretory vesicles is thought to continue throughout the remainder of oocyte development through both the in vivo synthesis of immature CA and their subsequent maturation into exocytotically competent CA [2, 3].

Teleost CA retain their evolutionarily conserved functions in the prevention of polyspermy [4]. However, unlike in more commonly studied mammalian and echinoderm models, fertilization of teleost eggs does not operate through the direct binding of sperm to the zona pellucida (ZP)/chorion. Instead, teleost fertilization is mechanically mediated through the micropyle, of which the egg typically possesses only one, or sometimes several as is the case in *Acipenseriformes* [5]. The micropyle is a cone-like opening in the chorion through which sperm must pass to gain access to the animal pole of the egg. This restricted access point to the teleost egg typically facilitates the successful passage of a single sperm, owing to physical size constraints [6]. Thus, the principal way in which teleost CA prevent polyspermy is by the attenuation of sperm motility and expulsion of excess sperm from the micropyle following fertilization [7, 8]. Teleost CA also play an important role in chorion hardening [9, 10], the separation of the micropyle from the animal pole [11], and the subsequent permanent occlusion of the micropyle [12, 13]. This contrasts with the function of cortical granules (homologs of teleost CA) in mammalian, echinoderm, and amphibian models, where the principal mechanism of CA-associated polyspermy prevention involves the direct modification of ZP proteins to either remove sperm-binding sites or otherwise render the ZP non-receptive to further sperm-egg interactions [14–18].

Given this divergence in the molecular functions of CA between teleosts and other animal models, it raises the question of whether teleost CA may have evolved additional functions that could support their diverse reproductive strategies.

Interestingly, it has been suggested that teleost CA may have adapted to act as reservoirs of proteins which, upon CA exostosis, establish an extracellular maternally inherited innate immune defense system. Many studies that suggest such a function propose that this innate immune defense is primarily mediated through the agglutination of bacterial threats by CA-derived lectins [19–24]. The proposition that CA may function as part of a maternally inherited innate immune defense system is particularly compelling in teleosts, as developing embryos are often subjected to a hostile external environment and therefore must rely on the hardened chorion and endogenous stores of protective biochemicals to regulate and maintain developmental competency.

While some isolated proteins such as lectins [20, 25–30], hyosophorins [31, 32], and enzymes [33], as well as other inorganic constituents, such as calcium and zinc [34, 35], have been identified as components of CA, little effort has been directed at developing a more comprehensive understanding of their proteomic composition. With growing evidence that teleost CA may be involved in a more diverse range of functions during early embryogenesis than initially appreciated, establishing a more thorough understanding of their proteomic composition will help to define the possible mechanisms through which CA-derived proteins may act to support both successful fertilization and subsequent embryonic development.

This study therefore aimed to identify the proteins which were proportionally over-represented in the zebrafish perivitelline fluid (PVF) shortly following CA exocytosis. Perivitelline fluid was extracted from water-activated zebrafish eggs following a non-destructive sampling technique which facilitated PVF, yolk/blastodisc/ooplasm (hereafter referred to simply as “yolk”), and chorion collection without the need for fixatives [36]. The use of fixatives during sample extraction can compromise the reliability of downstream mass spectrometry-based analyses [37–40]. This issue is of particular concern when investigating a largely uncharacterized proteome, such as that of the early zebrafish PVF, where fixation-induced artifacts may result in the underrepresentation of novel and/or biologically relevant proteins. While protocols have been developed to mitigate the impact of formalin fixation on proteomic analyses [41–44], these approaches require additional recovery steps and may still result in reduced sensitivity relative to the gold standard of fresh-frozen samples [37]. Consequently, the collection of fresh PVF from zebrafish eggs provides a straightforward and reliable means of overcoming this limitation.

A comparative analysis was performed between the PVF and the yolk proteomes to identify proteins which were over-represented in the PVF. Identifying the PVF proteome immediately following CA-exocytosis represents a critical step toward understanding the composition of teleost CA and, more broadly, enhances our understanding of the maternal investments that support embryogenesis. Elucidating the composition of the extracellular microenvironment of fish eggs is also an essential step toward understanding how fish embryos regulate their interactions with the external microenvironment throughout development and may provide novel insights into new methods and/or therapeutics which could be leveraged to enhance the hatching rates of farmed fish.

## Methods

### Animals

Mixed-sex zebrafish (*Danio rerio*; AB strain) were housed in a recirculating system which was maintained at 25°C. Fish were subject to a 13.5-h light cycle, with a 30-min ramp up and down of light intensity to simulate dawn and dusk, respectively. Fish were fed once daily to satiation with protein-based granules (ZM-400; ZM-Fish Food and Equipment, Winchester, UK). All animal handling and euthanasia procedures were approved in accordance with the standards of the Animal Ethics Committee for the University of Otago.

### Egg collection

Egg collection methods are described in Lewis et al. [36]. Briefly, ovulation was stimulated by transferring one female and two male fish into an optically translucent and water permeable segregated breeding tank (Tecniplast, Buguggiate, Italy) the day before egg collection. The following morning, the female fish was netted from the spawning tank and euthanized by submersion in a lethal overdose of 250 mg/L of benzocaine. Following euthanasia, fish were blotted dry, and the eggs were manually stripped. Only egg batches which exhibited characteristically healthy traits, such as optical translucency and a low prevalence of atretic oocytes [45], were used for this experiment. Stripped eggs were immediately transferred into pre-warmed (23°C) salmon ovarian fluid (collected from spawning chinook salmon, *Oncorhynchus tshawytscha*, and stored frozen, as per Johnson et al. [46]), to prevent premature egg activation [47, 48]. The eggs remained in this fluid at 23°C until they were used for sample collection; this lag period was no longer than 1.5 h.

### Collection of perivitelline fluid

Collection of PVF was undertaken exactly as described in Lewis et al. [36]. In brief, PVF was aspirated from live water-activated eggs by inserting a glass microneedle into the perivitelline space (PVS) and using the “fill” function on a microinjector (Nanoject II; Drummond Scientific Company, Broomall, US). PVF samples were snap frozen on dry ice and stored at −70°C until use. Between 30 and 50 μL of PVF was collected per fish, cumulatively derived from approximately 70 eggs per fish.

### Collection of chorions and yolk

Following PVF collection, aspirated eggs were transferred to a petri dish with fresh Milli-Q ultrapure water and their chorions were manually removed using a shearing action with two fine tipped forceps. This both removed the chorion and freed the remainder of the dechorionated egg which consisted predominantly of yolk, ooplasm, and blastodisc (referred to as “yolk”). Chorions were transferred to a 72-well microplate containing Hank balanced salt solution with 0.05% Tween (HBSS-T). The yolk was cleaned of any remaining PVF by two partial changes with fresh Milli-Q water. Yolk samples were snap frozen on dry ice and stored at −70°C.

Chorions were washed six times with HBSS-T followed by a final rinse in Milli-Q water. They were mechanically agitated for 30 s with forceps during each washing step to remove any yolk or PVF contaminants. Washed chorions were subsequently snap-frozen on dry ice and stored at −70°C.

Samples from individual fish were stored and analyzed separately with approximately 30 chorions and their respective yolk bodies pooled per fish.

### Mass spectrometry protein identification

#### Sample processing

Samples were collected from the ovulated eggs of three adult zebrafish. Accordingly, PVF and yolk protein samples consisted of three biological replicates each. Chorion protein data were derived from two biological replicates, owing to an insufficient protein yield from one fish.

Samples were sent to the Centre for Protein Research (Division of Health Sciences, University of Otago) for protein identification via mass spectrometry-based shotgun proteomics. All samples were cleaned and subjected to tryptic digestion using S-Trap mini spin columns (ProtiFi, Fairport, US), according to the manufacturer’s instructions. Following digestion, a preliminary liquid chromatography–tandem mass spectrometry (LC–MS/MS) run was performed for all samples to estimate total ion count (TIC) intensities. Total ion count values were used to assess relative peptide signal abundance across samples. Most samples displayed comparable TIC intensities; however, one chorion sample exhibited substantially lower signal. For final LC–MS/MS analyses, injection volumes were adjusted based on TIC values to normalize peptide loading across the comparable samples, which were analyzed using a 120-min gradient. The low-intensity chorion sample was analyzed using the maximum available digest volume and a shorter 40-min gradient. Samples were analyzed with triplicate technical replicates. Peptides were analyzed using liquid chromatography-mass spectrometry-based protein profiling with a linear trap quadrupole Orbitrap XL mass spectrometer, coupled in-line with an Ultimate 3000 nano-flow ultra-high performance liquid chromatography system (Thermo Fisher, Waltham, US). Three blank runs were performed in between each sample to avoid sample carryover. Peptides were identified against UniProt zebrafish protein database (Proteome ID, UP000000437) utilizing the SEQUEST search engine for sequence database-dependent analysis. A false discovery rate (FDR) threshold of 0.01 was set, based on a decoy database search. The mass spectrometry proteomics data have been deposited to the ProteomeXchange Consortium via the PRIDE partner repository (www.ebi.ac.uk/pride) with the dataset identifier PXD073748.

### Protein analysis

Relative abundance ratios were derived to identify proteins which were proportionally over-represented in the PVF relative to the yolk. Chorion samples were not used for comparative proteomic analysis. Averages were calculated from biological replicates, each of which was derived from the mean of its respective technical replicates. Unpaired Welch T-tests followed by two-stage step-up FDR (FDR = 0.05) assessment [49] were used to ascertain statistically significant variations in relative abundance between protein samples (*n* = 3, GraphPad Prism, version: 10.3.1).

Proteins that were over-represented in the PVF relative to yolk (Supplementary Files S2 and S3) were subject to a preliminary functional analysis by testing the statistical over-representation of molecular functions using the ShinyGO software (version: 0.80 [50]). Molecular functions were derived from associated gene ontology annotations. An FDR of 0.01 was used as cut-off for significant over-representation and only those molecular functions represented by at least two unique proteins in the dataset were included. Perivitelline fluid protein over-representation was assessed against a background dataset comprised of all zebrafish proteins identified by this project (*n* = 485). Protein hits were translated into their respective Zebrafish Information Network (ZFIN) gene identifiers for compatibility with ShinyGO. In circumstances where ZFIN gene IDs could not be attained, Ensembl gene IDs were sought. Compatible gene identifiers could not be assigned to 16 proteins in the over-represented PVF dataset and 46 proteins in the background dataset.

General proteomic compositions of the protein samples are graphically portrayed at the level of protein families and were derived from all proteins which individually constitute at least 0.05% of the average protein abundance. This cut-off represents 98.66%, 94.35%, 98.41%, of the average total protein abundance in the chorion, yolk, and PVF, respectively. Where a protein’s family was not identified in UniProt, the respective FASTA sequence was searched using InterPro's protein signature database (www.ebi.ac.uk/interpro) and the National Center for Biotechnology Information's (NCBI) protein Basic Local Alignment Search Tool (BLAST, blast.ncbi.nlm.nih.gov) to identify an appropriate family. Proteins for which family classification could not be determined were labeled as “uncharacterized.” For ease of graphical interpretation, protein families that did not constitute at least 1% of the total protein abundance, or did not meet the aforementioned cut-off criteria, were labeled as “other.”

The protein abundance heat map and principal component analysis plot were created using proteome discoverer software (Thermo Fisher, version 3.1). Both the heat map and principal component analysis were derived from protein abundance data from the entire mass spectrometry dataset. Euclidean distance with complete linkage was used to calculate the hierarchical clusters for the heat map. The protein abundance data were scaled prior to clustering.

## Results

### Mass spectrometry protein identification

In total, 485 unique proteins were identified across all samples, with 419 identified in chorion samples, 460 identified in the PVF, and 466 proteins identified in yolk (Supplementary File S1).

### Proteomic composition of the perivitelline fluid

**Figure 1:**
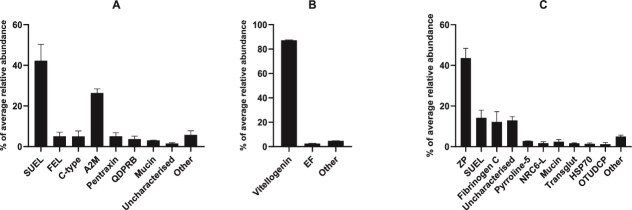
The average relative abundance of protein families as identified in the zebrafish (*Danio rerio*) perivitelline fluid (A), the yolk (B), and the chorion (C). Averages were calculated from biological replicates, each of which was derived from the mean of its respective technical replicates. All proteins in the “other” category represent protein groups which did not constitute at least 1% of the average total protein abundance. SUEL = sea urchin egg lectin-like lectins, A2M = alpha-2-macroglobulins, FEL = fish egg lectin, C-type = C-type lectin, QDPRB = quinoid dihydropteridine reductase b, EF = elongation factor, ZP = zona pellucida, NRC-6-L = nuclear receptor coactivator 6-like, Transglut = transglutaminase, HSP70 = heat shock protein 70, OTUDCP = ovarian tumour domain-containing protein 4. Bars represent mean percentage of total protein abundance; error bars represent standard deviations.

The PVF was predominantly composed of lectins, making up an average of 51.98% (±3.57%) of the average total protein abundance (Figure 1A). This lectin component was derived from three major protein families: sea urchin egg lectin-like (SUEL-like) lectins, fish egg lectins (FELs), and C-type lectins (Figure 1A). SUEL-like lectins comprised the majority of the lectin component, represented by 17 proteins within the given abundance parameters, whereas C-type lectins and FELs were represented by three and two proteins, respectively.

Other major protein classes represented in the PVF include alpha-2-macroglobulins (A2Ms), pentraxins, mucins, and dihydropteridine reductases (Figure 1A).

The A2Ms identified in the PVF can be split into two major sub-families: alpha-2-macroglobulin-like (A2M-L) proteins and pregnancy zone proteins (PZPs), which together constituted 26.28% (±1.67%) of the average total protein abundance. Five A2M-L proteins were identified within the PVF, which constituted an average of 13.86% (±1.76%) of the total protein abundance. Three PZPs were identified in the PVF, the most abundant of which corresponded to the highest abundance protein from the A2M family, individually constituting an average of 9.33% (±1%) of the total protein abundance. The three identified PZPs amounted to 12.42% (±0.95%) of the average total protein abundance.

Pentraxins and mucins were represented by a single member each, C-reactive protein-2 (CRP-2) and mucin-5AC isoform X1, which constituted an average of 5.03% (±1.44%) and 2.98% (±0.14%) of the total protein abundance, respectively.

Finally, four members of the dihydropteridine reductase protein family made up 3.68% (±1.15%) of the average PVF total protein abundance.

### Proteomic composition of the yolk

Yolk samples were predominantly composed of vitellogenin (VTG) proteins (Figure 1B). VTG 1–7 were identified in the zebrafish yolk samples. Two isoforms of both VTG-2 and VTG-7 were identified. These VTGs constituted 87.19% (±0.32%) of the total protein abundance, on average. The single most abundant non-VTG protein identified was eukaryotic translation elongation factor 2 (2.11% ±0.07%, average total protein abundance), followed by alpha-actinin-4 (0.31% ±0.04%, average total protein abundance). The only other protein family which met the threshold criteria (see the “Protein analysis” section under “Methods” for details) was elongation factor proteins (Figure 1B). Within the confines of these requirements, the elongation factor protein family was represented by four individual proteins which, when combined, totaled 2.46% (±0.16) of the average total protein abundance. The non-VTG proteomic composition of the yolk consisted of a large number of relatively low abundant proteins (Supplementary File S1).

### Proteomic composition of the chorion

The chorion was predominantly composed of ZP proteins, comprising 42.67% (±5.27) of the average total protein abundance (Figure 1C) with the single most abundant ZP protein being ZP2a at 14.73% (±3.13), while ZP2, ZP3, and to a lesser extent ZP4, were also identified in the zebrafish chorion. Additionally, several uncharacterized proteins were detected which when searched against the InterPro protein classification algorithm were identified as having ZP domains. Approximately 43% of the ZP proteins identified belonged to the ZP3 family, while 36%, 21%, and 0.02% of the ZP proteins were represented by ZP2, uncharacterized, and ZP4 proteins, respectively.

The second most abundant protein family identified in the chorion proteome was SUEL-like lectins, which made up 13.85% (±3.61%) of the average total protein abundance (Figure 1C).

Proteins belonging to the fibrinogen C-terminal domain-containing protein family were the third most abundant non-ZP protein family represented in chorion samples, constituting an average of 11.86% (±4.93%) of the total protein abundance (Figure 1C). This protein family was represented by two individual proteins, both of which represented the two most abundant non-ZP proteins identified in chorion samples. These proteins are derived from two transcript variants of a single gene (*microfibril associated protein 4, tandem duplicate 2*) which codes for microfibril-associated glycoprotein 4-like proteins.

Pyrroline-5-carboxylate reductase, nuclear receptor coactivator 6-like, heat shock protein 70, ovarian tumour domain-containing protein 4, and transglutaminase families were each represented by a single relatively highly abundant (>1%) protein. Finally, the mucin protein family was represented by six members, including three mucin-5AC isoforms and individual isoforms of mucins 2, 17, and 19, constituting a total of 2.36% (±1.10%) of the average relative protein abundance. The most abundant mucin sub-type was mucin-19, constituting an average of 1.1% (±0.37%) of the relative protein abundance.

### Proteomic comparisons

**Figure 2:**
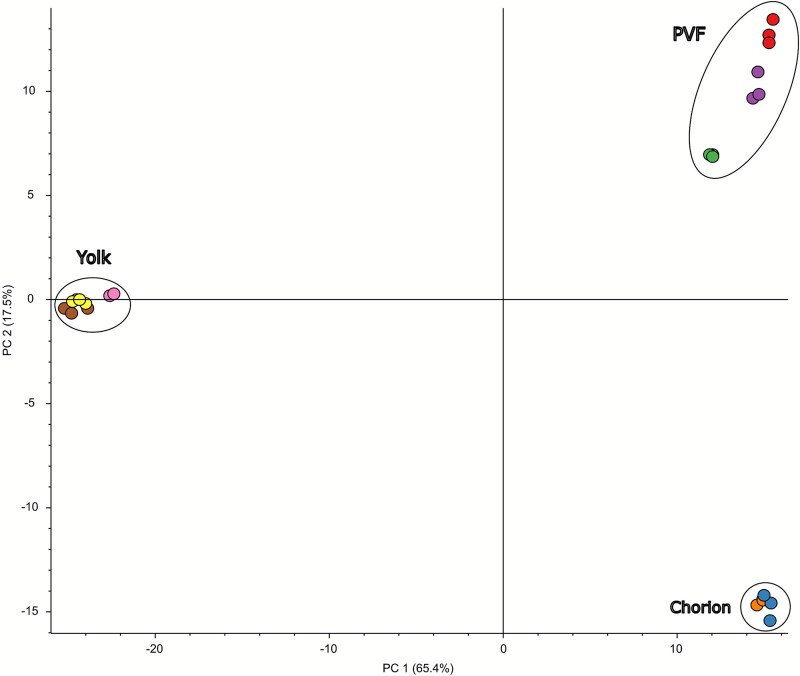
A principal component analysis grouped biological replicates of all protein samples with regards to protein abundance. Each biological replicate was run with three technical replicates. Technical replicates for each biological replicate are depicted as circles of the same color.

**Figure 3:**
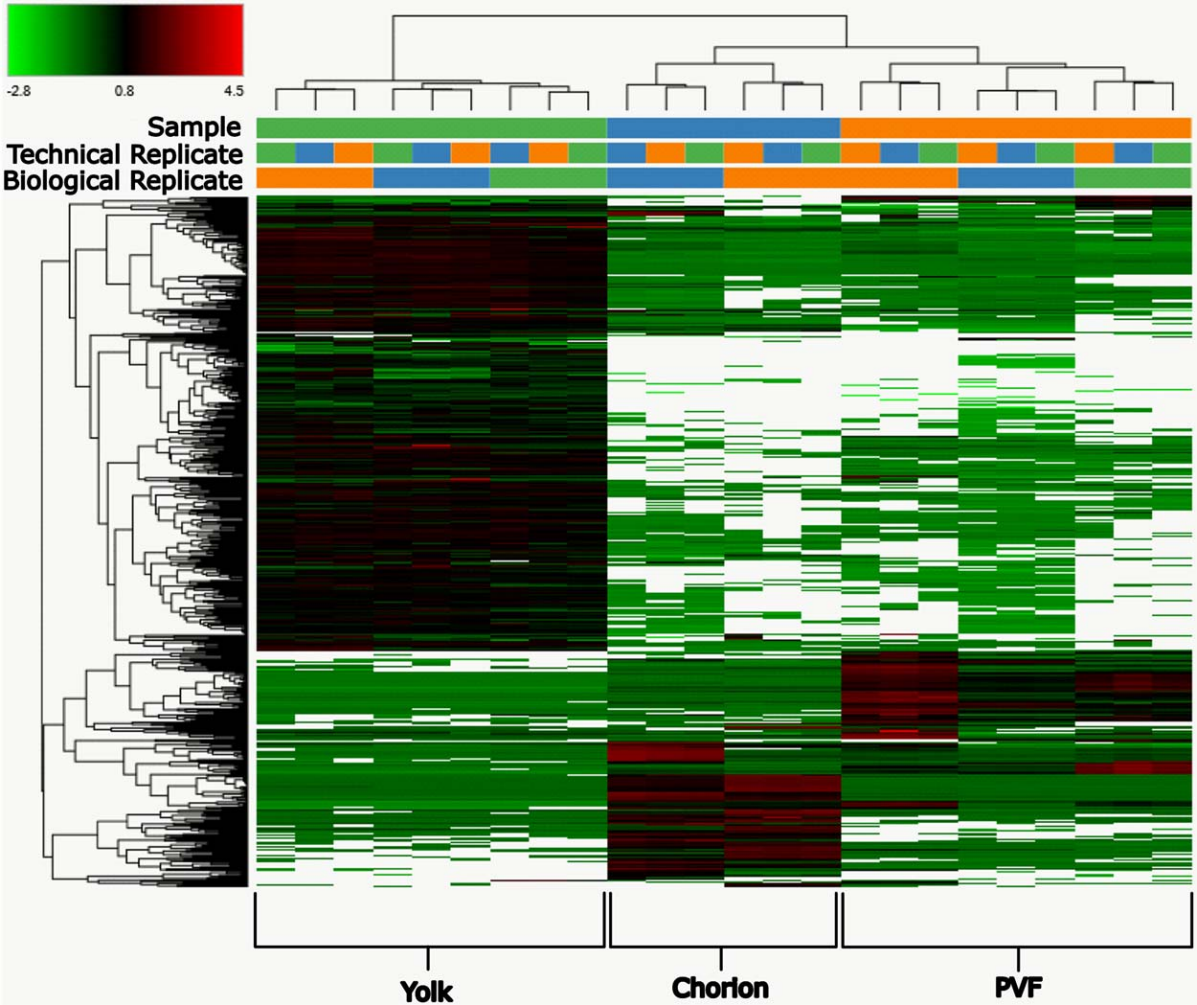
A heatmap of all proteins identified by this study among all biological samples. The green-red color gradient represents differential protein abundance between biological replicates. White represents proteins which were not identified within a particular sample. Sample types, biological replicates, and technical replicates are all represented. PVF = perivitelline fluid.

A principal component analysis overtly grouped all biological replicates into their respective samples of origin (Figure 2). PC1 and PC2 cumulatively explain 82.9% of the variance in the protein abundance data. This distinctiveness of the protein profiles between sample types is further validated in Figure 3, where the heat map depicts unique clusters of highly abundant proteins identified within yolk, chorion, and PVF samples.

**Figure 4:**
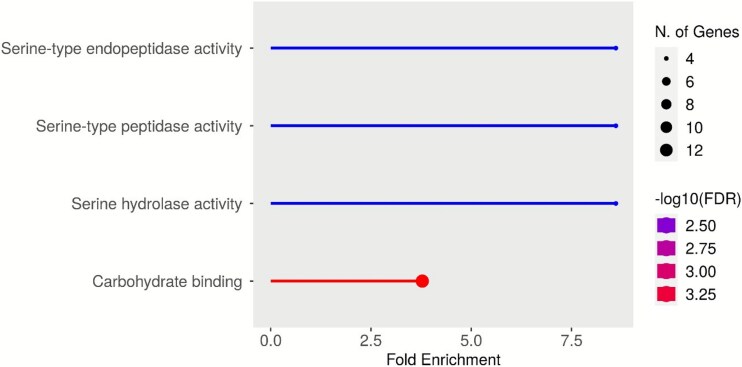
The fold enrichment of molecular functions associated with proportionally over-represented perivitelline fluid proteins relative to the general proteomic composition of the zebrafish (*Danio rerio*) egg as identified by this study. The purple-red color scale represents −log 10(FDR), while the lollypop head size represents how many genes (representative of proteins) are associated with that particular molecular function. Fold enrichment of molecular functions was assessed by the ShinyGO software (version:0.80 [50]).

The 44 proteins proportionally over-represented in the PVF (Supplementary Files S2 and S3), relative to the yolk, showed significantly enriched molecular functions associated with carbohydrate binding and broad-spectrum endopeptidase inhibitor activity, compared to all other zebrafish egg proteins identified in this study (Figure 4). Carbohydrate-binding-associated proteins were 3.8-fold enriched in the PVF, whereas all enriched endopeptidase regulatory-related functions were characterized by an 8.6-fold enrichment. Carbohydrate-binding functions were represented by 11 proteins, eight of which belonged to the SUEL-like lectin family. A single C-type lectin, an alpha-mannosidase, and an uncharacterized protein were also associated with the enrichment of carbohydrate-binding functions.

Enzyme regulator functions have been split into three separate groups by ShinyGO. All three groups were represented by the same four A2M-like proteins.

## Discussion

There is growing evidence to suggest that teleost CA facilitate more diverse biological roles throughout fertilization and embryogenesis than currently appreciated. For this reason, this study aimed to identify candidate CA-related proteins through characterization of the proteins which were over-represented in the PVF immediately following CA-exocytosis.

This study utilized a non-destructive and sensitive technique for the extraction of PVF following water-activation of the zebrafish egg [36]. This technique eliminated the necessity for protein fixation prior to sample collection, a step which was previously required for obtaining PVF from early-stage zebrafish eggs and embryos [24], resulting in high-quality fresh-frozen samples for use in downstream mass spectrometry analysis. Protein fixation is known to hinder mass-spectrometry–based protein identification, as fixation-induced crosslinking can obscure peptide detection and compromise sequence identification [37, 39, 40]. By avoiding fixation, this study was able to identify novel proteins present within the zebrafish PVF and provide new evidence for the maternal origin of PVF proteins that were previously thought to be produced by the embryo. Together, these advances offer the most robust characterization of the maternal contributions to the zebrafish PVF to date.

It is possible that the extremely high abundance of VTGs in yolk samples obscured the detection of lower abundance proteins and thus may have contributed to an over-estimation of the number of proteins identified as being over-represented in the PVF relative to yolk. While this should be taken into consideration, it does not alter the observation that the proteins identified as being over-represented were consistently found in the PVF following CA exocytosis. Importantly, the comparative analysis provides a practical means of narrowing the focus to proteins with consistent representation within the PVF, while excluding most proteins that are likely to reflect contamination from the yolk fraction. To contextualize the level of cross-sample contamination, VTGs made up approximately 87.8% of the total protein abundance in yolk samples, whereas they constituted approximately 0.1% and 0.5% of the average total protein abundance in PVF and chorion samples, respectively. This example highlights the relatively low level of yolk-derived contamination present in the PVF samples and reinforces the utility of the applied methodology for non-destructive PVF extraction.

While the pre-activation intracellular localization(s) of the proteins identified by this study remain unknown, it is reasonable to assume that many of the proteins present in the over-represented PVF proteome are of CA origin. While some proteins have been identified as being present in the PVS prior to egg activation, such as ovochymase in xenopus [51], the majority of proteins present in the PVF of teleosts directly following egg activation are thought to be derived from CA [24].

A list of 44 proteins was proportionally over-represented in the PVF. Analysis of the over-represented molecular functions of this list revealed an enrichment of proteins which are associated with carbohydrate binding and enzyme regulation. These functions were predominantly associated with lectins and A2M proteins, respectively.

The subsequent discussion examines the composition of the over-represented PVF proteins and their possible functions. It should be noted that these functions remain speculative and are inferred from their roles in other biological systems. Further experimental validation is necessary to definitively elucidate the functions of these proteins in the zebrafish PVF.

### SUEL-like lectins

SUEL-like lectins were the single most abundant component of the zebrafish PVF constituting 17 out of the 44 proteins identified in the over-represented protein dataset. SUEL-like lectins have been identified as major constituents in the eggs of *Salvelinus leucomaenis* (whitespotted char [52]), *Plecoglossus altivelis* (ayu sweetfish [53], *Oncorhynchus rhodurus* (Biwa trout [54]), *Ctenopharyngodon idella* (grass carp [55]), *Tribolodon brandtii* (Pacific redfin [53]), *Silurus asotus* (Amur catfish [56]), and *D. rerio* (zebrafish [24]), and have been specifically localized to the CA of *Oncorhynchus mykiss* (rainbow trout [57]), *O. tshawytscha* (Chinook salmon [29])*, Perca fluviatilis* (European perch), and *Tinca tinca* (Tench [27]) oocytes/eggs.

Members of the SUEL-like lectin family have a diverse array of carbohydrate affinities, dimerization configurations, tissue expression patterns, and functional roles [58]. As a result of their diversity, SUEL-like lectins have been implicated in sperm agglutination [59], pathogen recognition and agglutination [19, 60, 61, 62], intra-ooplasmic CA organization [25], inflammation regulation [63], and the modification of the extraembryonic matrix [64]. The ubiquitous affinity of SUEL-like lectins for L-rhamnose is particularly noteworthy in the context of their proposed role in bacterial agglutination, given that L-rhamnose rarely occurs in the Animal Kingdom, yet is a common and essential component of both gram negative and gram-positive bacterial cell walls [65–67].

Interestingly, SUEL-like lectins were identified in high abundances in chorion samples (Figure 1C). The zebrafish chorion is known to be glycosylated with beta-D-galactose, to which SUEL-like lectins frequently exhibit a secondary affinity [68]. Therefore, it is possible that SUEL-like lectins in the PVF may migrate with the distending chorion through their affinity to D-galactose. Cortical alveoli-derived SUEL-like lectins have been demonstrated to interact with the chorion in Rainbow trout (*O. mykiss*) through their associations with both the surface of the ooplasm and the inside of the chorion post-egg-activation [66]. Moreover, in activated perch (*P. fluviatilis*) eggs, CA-derived lectins have been shown to retain excess carbohydrate-binding activity following their migration to the external egg jelly coat post-activation [26]. This retained binding activity of the migrated lectins is thought to function as an innate immune defense barrier for the developing embryo through their ability to agglutinate any bacteria that come into contact with the jelly coat [26].

### C-type lectins, fish egg lectins, pentraxins

C-type lectins, FELs, and pentraxins comprised the other major families of lectins identified as being over-represented in the PVF. C-type lectins are a family of Ca^2+^-dependent proteins that share structural homology in their carbohydrate-binding domains yet exhibit a diverse range of binding affinities, molecular interactions, and biological functions [60].

C-type lectins have been identified as components of teleost oocytes in *Osmerus lanceolatus* [69] and *D. rerio* [24] and have been specifically localized to CA in *Carassius gibelio* oocytes [20]. Similar to SUEL-like lectins, C-type lectins have been widely implicated in innate immunity, predominantly with respect to their ability to agglutinate bacteria [70–73]. However, some members have also been demonstrated to bind to sugars on the external surfaces of fungi, parasites, and viruses [59, 61].

Fish-egg-lectins display high homology to the tachylectin family, which are involved in innate immune system function in arthropods [62–64] and have been identified as components of fish eggs in *Cyprinus carpio* [63] and *D. rerio* [68].

The specific FEL which has been identified as over-represented in the PVF by this study has been previously identified as a maternally derived component of the zebrafish egg, although its intracellular localization within the egg was not known at the time [68]. It was demonstrated that the zebrafish FEL acts as an opsonin and can interact with both gram-positive and -negative bacteria [68]. Moreover, increased survival rates were observed in FEL-injected embryos which were subject to bacterial attack [68].

Interestingly, previous studies did not observe the presence of FELs in the PVS of zebrafish eggs until 2 days post-fertilization [24]. While not explicitly stated, this result leads to the conclusion that FELs are produced by the embryo, rather than being maternally inherited. Contrary to this, our study identified a relatively high abundance of FELs in the PVS directly following egg activation, revealing that FELs are a maternally provided component of the zebrafish PVF.

The final major lectin-like family over-represented in the PVF protein fraction is pentraxins. This family is exclusively represented in the PVF by C-reactive protein-2 (CRP/CRP2). While pentraxins do not strictly fall under the banner of “lectins,” in the present context they function in similar ways by acting as pattern recognition proteins of the innate immune system and are therefore discussed herein [74]. C-reactive proteins have been identified as components in the eggs of *Takifugu rubripes* [75], *Cyclopterus lumpus* [76], and *D. rerio* [24]. Pentraxins play a broad role in innate immunity with CRP being first identified for its ability to bind C-polysaccharide and phosphorylcholine, which are present on the surface of both apoptotic eukaryotic cells and bacterial cell walls [70, 71].

Multiple CRP genes have emerged in zebrafish, exhibiting heterogeneous expression in response to viral infection [72]. This variability in their expression patterns suggests that CRPs in zebrafish may have acquired novel functions. Interestingly, when the adaptive immune system is inhibited in zebrafish, all CRPs, apart from CRP2, are upregulated in response to viral infection [72]. This is contrasted by CRP2 and CRP3 being the only CRPs to be upregulated in response to bacterial infection of zebrafish embryos [73].

### Alpha-2-macroglobulins

The second most abundant protein family represented in the over-represented PVF protein dataset was A2M proteins. This protein family was represented by two sub-families in the PVF: PZPs and A2M-Ls. Both PZPs and A2M-L proteins are heavily glycosylated broad spectrum protease inhibitors, although, A2Ms display far broader protease inhibition than PZPs [77]. The predominant function of PZP in mammals is the immunosuppression of T-cell function to prevent fetal rejection during late-term pregnancy. It has also been shown to function as a chaperone with regards to the clearance of misfolded proteins in the placenta [78–80]. As most teleosts are external spawners, it is unlikely that this specific biological function is also present, though they may still function as protein chaperones in a different context. A2Ms are typically expressed in the liver and distributed throughout the plasma where they function as components of the innate immune system [81]. However, their presence in eggs has also previously been reported in *Gallus domesticus* [82], *Branchiostoma japonicum* [4, 8], and *D. rerio* [24]. The biological role of PZPs in teleosts is not yet clear, although they likely maintain similar molecular functions in proteinase inhibition as observed in mammalian models [83].

A2Ms are widely implicated in the functioning of the innate immune system by their ability to trap, inhibit, and subsequently clear a diverse array of peptidases [84, 85]. This broad peptidase regulation facilitates the ability of A2Ms to attenuate the impacts of pathogenic attacks via counteraction of bacterial and parasitic peptidases [84, 86, 87]. Given their extensive association with innate immunity, it is possible that the enriched A2M proteins in the PVF confer a secondary line of defense against peptidases which make their way through the chorion. Alternatively, A2Ms may play a role in regulating native peptidases within the PVF.

### Other proteins of interest

Several other lower abundance proteins were identified as being over-represented in the PVF. While less abundant than the other major protein families, their presence in the PVF has interesting functional implications.

#### Metalloprotease

A single astacin-like metalloprotease was identified in the PVF. This is particularly noteworthy, as an astacin metalloprotease, termed alveolin, has been identified as the key CA-derived protease responsible for the initiation of chorion hardening in *Oryzias latipes* (medaka; [10]). Alveolin-initiated chorion hardening is facilitated by its ability to both partially cleave ZP proteins as well as process and to activate the chorion-derived transglutaminase responsible for the subsequent ZP peptide crosslinking upon egg activation [9, 10]. In a phylogenetic analysis of the molecular evolution of alveolin, a zebrafish metalloprotease protein, zinc metalloproteinase nas-15-like isoform 1 (NCBI reference sequence: XP_001337538.3), was identified as a potential homolog of alveolin owing to their high sequence similarities [33]. The original zebrafish protein identified as being homologous with medaka alveolin has since been superseded in the NCBI protein database by the same astacin-like metalloprotease identified in this study (UniProt accession: A0A8M6Z8J3; NCBI reference sequence: NP_001373298.1). While the same metalloprotease has been previously identified in the PVF of zebrafish embryos [24], this study is the first to report its maternal origin. The close evolutionary relatedness to medaka alveolin and its maternal origin in the PVF shortly following CA exocytosis makes the identified metalloprotease a compelling candidate for the zebrafish homolog of medaka alveolin.

#### Peptide:N-glycosidase F

Peptide:N-glycosidase F (PNGase F) is an amidase that catalyzes the removal of N-linked oligosaccharides from glycoproteins [88]. Peptide:N-glycosidases have been implicated in the removal of N-linked glycans from yolk glycoproteins in medaka eggs, as well as the liberation of glycans from a CA-derived polysialoglycoprotein, termed hyosophorin, in medaka embryos [31, 89].

Zebrafish CA are rich in carbohydrates, based on strong positive periodic acid–Schiff staining [36]. While anticipating the functions of PNGase F in the zebrafish PVF remains speculative, it may act to liberate carbohydrates from CA-derived glycoproteins thereby increasing the osmotic gradient of the PVS and facilitating diffusion of water across the chorion. It is also possible that PNGase F may act to post translationally alter the functions of glycoproteins in the PVF [31].

#### Ovochymase

Ovochymase is a chymotrypsin-like protease which has been identified within the preactivated PVS of Xenopus eggs [90, 91]. These proteases are present in the Xenopus PVS as proenzymes which are transiently activated by a trypsin-like protease in a stage-specific manner during early embryogenesis [92]. Its presence within the PVS prior to cortical granule exocytosis precludes its presence in cortical granules in Xenopus. However, its activation by a trypsin-like protease upon egg activation suggest that there may be an interaction between ovochymase and a cortical granule-derived protease [51, 93, 94]. Very little is known regarding the function of ovochymase in fish eggs. In fact, to the best of our knowledge, there is no explicit mention of the presence of ovochymase in fish eggs within the current literature, although it was identified in passing as a component of the PVF in zebrafish [24]. Interestingly, it was only identified in the PVS directly following fertilization and was not identified during later stages of embryogenesis [24]. This suggests that ovochymase plays a stage specific role, potentially in coordination with CA-exocytosis, during the initial stages of fertilization/embryogenesis.

### Chorion proteomic analysis

Due to the low statistical power resulting from the limited sample size of chorions, statistically rigorous comparisons between the protein composition of the PVF and chorion were not possible. However, interesting observations can still be drawn from this dataset.

The chorion is predominantly composed of ZP proteins; however, their relative abundance is surprisingly low, comprising only 45.9% (±8.97) of the average total protein abundance. This relatively low abundance may be explained by two non-mutually exclusive theories. As chorions were collected approximately 20 min post-egg-activation, ZP proteins would have already undergone modifications associated with the “chorion hardening” process. Zona pellucida protein restructuring both reduces the solubility of the ZP proteins and confers enzymatic resistance to the chorion [95–97]. The insoluble nature of ZP proteins post egg-activation may have resulted in a disproportionate amount of ZP proteins being removed from the sample during the pelleting of insoluble material prior to mass spectrometry analysis. Additionally, the enzymatic resistance conferred to the chorion post-egg-activation may have hindered the tryptic cleavage of the retained ZP proteins prior to peptide fingerprinting.

## Concluding remarks

This study utilized proteomic comparisons between the PVF and the yolk to identify proteins which were proportionally over-represented in the PVF following CA exocytosis. A total of 44 proteins were found to be over-represented in the PVF, many of which are known to play roles in innate immunity. Furthermore, this study provides strong evidence for the maternal origin of FELs, PNGase F, and a metalloprotease that represents a compelling candidate for a homolog of medaka alveolin, in the PVF of zebrafish eggs. While the intracellular localization of these proteins prior to egg activation remains unknown, given that PVF samples were taken shortly after CA exocytosis, it is probable that many of the most abundant proteins present in the over-represented PVF proteome are derived from CA. Establishing this over-represented proteomic profile of the PVF not only advances our understanding of the teleost CA proteome but also provides broader insights into the maternal contributions to the PVF. Future studies should aim to definitively determine the intracellular localization of putative CA proteins prior to CA exocytosis. Moreover, investigating the in vivo distribution of CA-related proteins both during oogenesis and after egg activation will provide valuable insights into CA biogenesis and their molecular functions within the PVF.

## Supplementary Material

Supp_1-3_All_Identified_Proteins_PVF_vs_Yolk_Over_represented_PVF_Proteins_ioag012

Supp_2_PVF_vs_Yolk_ioag012

Supp_3_Over-represented_PVF_proteins_ioag012

## Data Availability

The data underlying this article are available on PRIDE at www.ebi.ac.uk/pride and can be accessed with the identifier PXD073748.
